# Lived Experiences of Self-Care in Older Adults with Lymphoma Undergoing Chemotherapy Treatments During the COVID-19 Pandemic

**DOI:** 10.3390/healthcare13020157

**Published:** 2025-01-15

**Authors:** Samonnan Thasaneesuwan, Kittikorn Nilmanat, Anuchit Maropi, Sudjit Sukrai, Margaret I. Fitch

**Affiliations:** 1 Faculty of Nursing, Prince of Songkla University, Songkhla 90110, Thailand; kittikorn.n@psu.ac.th; 2 Faculty of Nursing, Princess of Naradhiwas University, Narathiwat 96000, Thailand; anuchit.m@pnu.ac.th; 3 Male Internal Medicine 3, Hat Yai Hospital, Songkhla 90110, Thailand; sudjit.kaew@hotmail.com; 4 Bloomberg Faculty of Nursing, University of Toronto, Toronto, ON M4C 4V9, Canada; marg.i.fitch@gmail.com

**Keywords:** self-care, qualitative research, older adults, cancer, COVID-19

## Abstract

**Background:** To be diagnosed and treated for cancer can be a distressing experience, and it can require individuals to engage in self-care strategies to cope with the situation. The self-care experiences of older adults with lymphoma undergoing chemotherapy during the COVID-19 pandemic in Thailand remain rarely studied. This study aimed to explore the lived experiences of self-care among older adults with lymphoma undergoing chemotherapy during the COVID-19 pandemic. **Methods:** A hermeneutic phenomenology research design was used. It involved in-depth interviews with nine older adults with lymphoma undergoing chemotherapy. Data analysis utilized van Manen’s approach, and trustworthiness was ensured by adhering to Lincoln and Guba’s criteria. **Results:** This study revealed that the lived experience of self-care among older adults with lymphoma receiving chemotherapy during the COVID-19 pandemic encompasses five lifeworlds within the lived body, addressing aspects of (1) thumjai and (2) continuing to fight cancer. Lived relations means trusting healthcare providers and holding on together. Lived time reflects being aware of the natural path of life. The concept of lived space encompasses staying safe at home and staying cautious. Lived things reflect (1) accepting one’s own karma and (2) living economically. **Conclusions:** The findings enhance the understanding of self-care experiences among older adults with lymphoma undergoing chemotherapy during the COVID-19 pandemic in an Asian context. The findings can inform the development of a person-centered nursing intervention tailored for older persons that emphasizes cultural aspects.

## 1. Introduction

The global population is aging, and due to this, the incidence of cancer in older adults is expected to increase [1]. According to Pilleron et al. [2], in 2020, there were more than six million new cancer cases diagnosed in adults aged 60 years and older living in low- and middle-income countries (LMICs), excluding non-melanoma skin cancer. If there is no change in the cancer risk pattern over the next several years, this population group is expected to reach 11.5 million in 2040 [2]. Hematological cancer is frequently reported among older adults, but the prognosis for this disease in individuals over 75 years of age is poor [3]. In Thailand, the number of new cancer cases has increased among older adults over 60 [4,5]. The mortality rates for hematologic cancer per 100,000 population between 2020 and 2022 were 4.220, 3.796, and 5.977, respectively [6]. Lymphoma ranks among the five most prevalent cancers in men, with the number of new cases rising to 2855 in 2017, 3016 in 2020, and 3476 in 2023 [7].

Care for older persons with cancer can be challenging [8,9]. It is often complicated by age-related vulnerabilities [10] and a high risk of patients suffering severe toxicities during chemotherapy treatment [11,12]. Older persons have reported difficulties in managing their cancer treatments and appointments, as well as financial hardship [13]. Compared to young persons, older individuals face a greater risk of adverse effects from cancer treatments, as well as the development or worsening of other health problems, which can result in declining health, chronic functional issues, and isolation from society [14]. In addition to cancer, older people may have at least one other chronic illness. Diabetes, COPD, depression, heart problems, and hypertension are the most common long-term illnesses among people living with and beyond cancer [15]. These conditions have a profound impact on the quality of life and mental well-being [11,15,16,17]. Prioritizing the interplay between age- and cancer-related issues before, during, and after cancer therapy is crucial for enhancing the prognosis and quality of life for older adult cancer survivors [18].

Self-care is crucial if individuals are to effectively manage chronic conditions [19]. Patients with cancer engage in self-care through a process that includes learning, self-monitoring, self-empowerment, and involvement in making decisions about their care [20]. A systematic review and synthesis of qualitative studies describing self-management found that older people attempted to manage their health conditions well to maintain their independent lives [21]. Rocha et al. [22] found that the concept of self-care in older persons with cancer was associated with alterations and modifications to their diets. However, they also worked to adapt to the changes emerging from the aging process and to the changes resulting from cancer and its treatment. Engaging in self-care may require new learning and access to additional resources on the part of older adults.

The COVID-19 pandemic significantly impacted lifestyles around the world, particularly for persons with cancer [23]. Individuals with cancer in all parts of the world tended to have greater emotional and financial difficulties than people with other chronic illnesses [24]. In Thailand, the impacts of COVID-19 on cancer care were reported by Sukhokanjanachusak [25]. Fear of contracting the disease led persons with cancer to postpone treatment appointments, resulting in an average delay in treatment of approximately 1.6–2 months. Consequently, the delay in treatment has contributed to heightened cancer morbidity and death. Due to low body immunity from disease pathology and chemotherapy adverse effects, COVID-19 was more severe in patients with hematological cancer and led to further fatalities [26]. Therefore, a study on self-care among persons with hematological cancer is essential.

To date, there has been insufficient research on the self-care experiences of older adults with hematological cancer undergoing chemotherapy in Thailand. Since the majority of patients in this group are older persons, and since Thailand is a completed aged society, understanding the self-care experiences of this group will help nurses to tailor person-centered care. This phenomenological study aimed to explore and describe the lived experiences of self-care in older adults with lymphoma who were undergoing chemotherapy treatments during the COVID-19 pandemic. The expected outcome is the implementation of appropriate interventions for vulnerable groups, such as advocating for policies in response to the emerging disease situation.

## 2. Materials and Methods

This study employed a hermeneutic phenomenology approach. Phenomenological research is the description of the lived experience and aims to gain a deeper understanding of the nature or meaning of everyday experiences [27,28]. The description and interpretation of the meanings an individual assigns to events in their lives leads to a depth and richness of understanding of their experiences.

This study took place at the tertiary hospital of Songkhla province. Data collection was conducted from November 2022 to April 2023. This period corresponded to the fifth wave of the COVID-19 pandemic, with the Omicron variant. Compared to the fourth wave (May 2021), the number of confirmed cases and deaths was slightly decreased. However, measures established in response to the COVID-19 epidemic remained strictly sustained. The Consolidated Criteria for Reporting Qualitative Research (COREQ) checklist was utilized for this qualitative report [29].

In the interest of data saturation, nine informants were purposively selected for participation in this study while being hospitalized. The inclusion criteria were as follows: (1) aged 60 years and above, (2) diagnosed with lymphoma, (3) received at least two cycles of chemotherapy, (4) fluent in Thai, (5) no cognitive impairment issues, (6) no evidence of depression, and (7) willing to participate in this study. Exclusion criteria included having severe fatigue preventing engagement in a continuous interview and immediate COVID-19 infection.

After gaining ethical approval from the Institutional Review Board (HYH EC 033-65-02), a nurse from the hematological unit helped with the recruitment process. She initially introduced the research project to the potential informants and asked about their interest in sharing their experiences. When potential informants showed their interest, the nurse contacted the first author about the consent process. Upon meeting the potential informants, the researcher explained the study objectives, data collection process, analysis, and ethical considerations. Those who agreed to participate signed a consent form and were asked for their preferred time and place for the interviews.

Data were gathered using an interview guide developed based on relevant literature reviews [20,30,31]. The interview script (questions) were reviewed by three experts in both qualitative research and cancer care. In addition, the interview guide was tested with two older persons. Subsequently, the main questions were modified to make better sense for the interview purposes.

The first author (ST) conducted all the interviews. She has a qualitative research background and has been involved in several qualitative research projects. The other three authors are also qualitative researchers with expertise in cancer care. Interviews were gathered in Thai starting with general topics such as personal information and current health concerns. After that, the PI started asking questions such as the following: “Could you share your concern related to COVID-19?”, “How did you feel?”, “How you perform self-care to prevent?”, “What were the hardships under the COVID-19 situation related with your disease?” and “Who supported you during the treatment?” During the interview, notes on key issues were taken by the interviewer for probing and follow-up questions. Observations were also noted regarding the participants’ facial expressions, posture, and behaviors. Interviews lasted 45 min on average (range 30 to 60 min) and were held 2–3 times per person, aiming to clarify emerging issues from previous interviews with that individual. All interviews were audio recorded and transcribed verbatim.

### 2.1. Trustworthiness

Several strategies were used to enhance trustworthiness [32]. Before the interview, the first author (ST) established a trusting relationship and ensured informants about the confidentiality of their data. In addition, the researcher facilitated a comfortable environment by listening attentively and providing opportunities for informants to make decisions to answer or omit the questions. Moreover, the author summarized the key points of each interview at the end of each session, aiming to verify the data with informants in future interviews. Furthermore, peer debriefing was used to mitigate bias. Regular dialogue was conducted among Thai researchers to validate and verify tentative analyses and findings, ensuring the confirmability of the results.

### 2.2. Data Analysis

The analysis was carried out concurrently with the gathering of data. The first and second authors (ST, KN) read and coded each transcript independently. Then, they shared their perspectives on emerging tentative codes and discussed any disagreement until consensus was reached. van Manen’ phenomenological approach was followed [27,28]. According to van Manen, the phenomenological analysis of human experiences consists of six steps, including (1) turning to the nature of lived experience, (2) investigating experience as we live it, (3) reflecting on essential themes, (4) writing and rewriting, (5) maintaining a strong and oriented relation, and (6) balancing and considering parts and the whole. In reflecting upon and uncovering the essential themes, the researcher used three approaches: (1) wholistic reading, (2) selective reading (listening and reading many times, including highlighting some of the statements), and (3) detailed reading (reading every single sentence and reflecting how the language depicts the phenomenon). In general, the transcripts were read and reread repeatedly, case by case, to maintain a connection between the field notes and the participant’s experience. Words and sentences with similar meanings were grouped into codes and codes with similar meanings were grouped into subthemes. These subthemes were then further organized into themes that represented the essence of lived experience. The five lifeworld existentials of van Manen were used to guide reflection in the later phase of data analysis [27,28]. These include the lived body (corporeality), lived self–other (relationality), lived time (temporality), lived space (spatiality), and lived thing (materiality), describing the way humans experience the world. Despite different points of focus, these five lifeworld existentials are intertwined and interact with one another [33].

## 3. Results

A total of nine older adults with lymphoma undergoing chemotherapy treatment participated in the in-depth interviews. The patients ranged from 62 to 72 years of age; six were male, and seven were married. The number of chemotherapy cycles they received ranged from 2 to 13. All but one had comorbidities (n = 8). All participants reported that they received care from at least one informal caregiver, often their children (n = 5) (Table 1).

### 3.1. Thematic Results

Guided by van Manen’s five lifeworld existentials, eight subthemes were extracted (Table 2). These lifeworld existentials and subthemes are intertwined.

### 3.2. Lived Body

#### 3.2.1. Thumjai

The word ‘thumjai’ was repeatedly expressed by several participants. These participants were hospitalized for intensive chemotherapy soon after a lymphoma diagnosis. They accepted that the moment of cancer disclosure from the doctor was a painful experience. However, they attempted to ‘thumjai’ or cope with being labeled as a person with cancer. As time went by, they gradually thumjai and lived their lives as normally as possible.

*It is natural. We have to thumjai. Don’t be stressful. Let’s say … everyone has the potential to get diseases, recover, and die. No one lives long-lasting. No matter how rich you are, you won’t last. Nothing to be fear of. Everyone was born and must die. We have to think what we live for. Live and then die. Happening, existing and ending … no need to be stressed on this matter. If not, you cannot live well. All diseases can recover … recover and die … those with diabetes die, with hypertension dies … those with cancer also die … all diseases lead to death… (P4)*

Based on the above quotation, this participant used Buddhist teaching on natural law to understand their circumstance. He examined what happened to him and related it to the Buddhist concept of impermanence. Similarly, another participant shared their experiences during chemotherapy treatments, when the cancer diagnosis and suffering from chemotherapy adverse effects discouraged her. She was overwhelmed by negative thoughts. Later on, she realized that there was no need to dwell on them. Therefore, she let the thoughts go and thumjai to accept her health condition and focus more on living.

*I thumjai. Don’t overthink … something like why we are like this … When we got this disease, we must thumjai. Although we are sick, we can live with it … I mean we thumjai that we got this disease. Don’t think too much that we got this disease … initially we might feel stress … I cried and thought too much … I was worried about my children … but after the cycle 3, I can thumjai … whatever will be, will be … if it is time to die, I will let it. Thumjai means don’t think too much … don’t think about the disease that we have … but let us think how to take care of ourselves and live well….that’s it. (P9)*

#### 3.2.2. Continuing to Fight Cancer

Participants stated that they had to fight for their survival. To achieve this mission, they tried to do several things, such as keeping their bodies strong and ready for chemotherapy. Most participants tried to improve their nutritional status by eating more food, while others focused more on exercise.

*Don’t think too much. Making our mind normalize. Thinking that we must keep fighting it. If we don’t eat, it is as if we give up life…it is like what people said ‘if we give up, we will get worse very soon.’ Because I want to fight, I try to eat plenty of foods. Although I don’t want to eat, I have to. I try to eat as much as I can. Recently, I eat a lot. It is like when we reach the point, we realized that if we sink in our thoughts, it would get worse. I just do our best at present. Make our body fit as it has been before. I will keep fighting until the end. (P1)*

Another participant explained that to keep on fighting cancer was considered self-reliance. She stated that her children had all grown up and left home. She had to take care of herself well, so that she would not depend on them and be a burden to them.

*I thought that if we don’t fight, who will fight for us. Some patients said that they felt discouraged. Why should we be discouraged? Nobody wants to get it (cancer), right? However, if we get it (cancer), we must keep fighting. If we don’t, who will? (P8)*

### 3.3. Lived Relation

#### 3.3.1. Trusting Healthcare Providers

Participants expressed that medical doctors and nurses were supportive people for them. Doctors and nurses always advised them on how to take care of themselves and how to monitor their symptoms. Participants trusted both medical doctors and nurses and adhered to their suggestions on self-care. They believed that if they followed what the doctors and nurses said strictly, their cancer could be cured.

*The doctor told me that it had the percentage of recovery is high. He said that many are cured and survive. The doctor mentioned that if the mass dose not grow, we can survive. The doctor suggested that if I find the nodule wherever, I must go to see him. Nurses provided good care for me too. Whatever they say, I do it. (P5)*

#### 3.3.2. Holding on Together

All participants stated that they received great support from their families, particularly their children. Although their children were married and had their own family, they frequently visited and took turns caring for them. Their family members were always by their side. One participant stated, ‘My son just came to talk with me. He bought food and put it in the refrigerator, and he went to work’. The participants acknowledged support and encouragement from their children and relatives. They indicated that encouragement was crucial during their treatment journey. Some of their family members provided financial support, while others prepared food for them. Another participant stated that her daughter visited and took care of her every evening when she received chemotherapy.

*Encouragement is important. People with this disease need encouragement, right? We need to be encouraged. If there is no encouragement, we will collapse quickly. Encouragement is important thing and the most important things are my children and husband. My children told me that I must fight, don’t worry … many other people got cancer too.…do not think about anything … no need to be stressful…when we stress, we will feel discomforted … so don’t be stress …. don’t think about negative things (P9)*

Furthermore, participants expressed concerns about their family. Participants also did not want their children and other family members to worry about them. The participants explained that the reason why they kept on fighting cancer was that they did not want to be a burden to their children.

*Encouragement or kumlungjai is powerful. I don’t want to get sick. I want to recover … but it is not (possible), particularly for this disease … it is just relieved … I must keep fighting … if I don’t, it will make my children upset … it is because if I feel blue and powerless, these negative energy will vibrate to surrounding people. (P8)*

### 3.4. Lived Time

#### Being Aware of the Natural Path of Life

The cancer diagnosis brought about thoughts related to life and death for the participants. Some participants said that living or dying is predetermined, yet the time frame of their lives was uncertain for them. Being aware of the natural path of life helped them accept the natural truth of life and accept what happens.

*Our lives are predetermined when we will go. As Buddhist monks said, we can determine birth, but we cannot determine death. When that time comes, you have to thumjai. So I don’t think about it. If it’s time to go, we have to go. No one is invincible. We are born and we must go whether it is slow or fast.*

Another participant expressed that he had reached his old age and had passed several challenges in his life. Therefore, there was nothing for him to be fearful of.

*We have to thumjai. Actually, people are at their age. Working age is 25–50. It is enjoyable. However, when it is 50–60, that is downhill. (I am) Coming to this age, (there is) no more fear. (P4)*

### 3.5. Lived Space

#### Staying Safe at Home and Staying Cautious

As this study was conducted during the fifth wave of COVID-19, participants were aware that they were at high risk of being infected with COVID-19. The risks were not only because of their older age, but also the effects of chemotherapy, as well as their comorbid diseases. As a result, they kept protecting themselves by social distancing and being cautious when going out. They always wore masks when they were in crowded places.

*Wherever I go, I always wear the mask. It prevents many diseases in the respiratory system … When going in the market or hospital, I wore a mask regularly. COVID is still there, so I have to take care of myself well. (P2)*

*I must be cautious, washing my hands, wearing masks when going out … I mainly stayed at home and asked my children to bring things to me. (P6)*

### 3.6. Lived Things

#### Accepting One’s Own Karma

Most participants often talked about their cancer experiences in relation to their karmic belief. They viewed cancer as a bad thing in their lives. It was clearly noticed that the participants never said the word cancer during the interviews. They used the word “it” instead. Some participants mentioned that the cause of their disease was due to their previous bad deeds. As a consequence, they had to pay the price of their previous karma.

*… I wondered if I had conducted a lot of karma. Then I got this disease because of karma? I had this idea in mind. (P1)*

*We must know what we have been doing during that time. Therefore, we must thumjai and not be stressed. If we survive, let us make merit. (P4)*

This karmic belief led the participants to perform religious practices. Participants made merit with the expectation of receiving good karmic recompense.

*Regarding this disease (cancer), if I recover, it is my merit … if not, it is my karma…let’s put it in this way … I am only wondering whether it recover or not … if it recover, that will be good. If it is not, it is up to karma … (P9)*

### 3.7. Living Economically

All but one of the participants were unemployed after retirement. However, they then worked in the informal labor sector with relatively low wages. Most participants experienced adverse effects from chemotherapy treatments. They reported a lack of energy to perform activities. In addition, they felt they became tired easily. As a result, they could not continue to work and earn money for a living.

*It gave me a hard time. I am sick. If I am well, I can earn money for 4–5 hundred or 800 baht. But now I can’t. I don’t have the energy. I’m upset that I can’t work. I’ve stopped working the rubber garden. (P1)*

*I have to eat rice with hot water. Take steamed rice and dissolve it with salt. The second cycle, it rained heavily, non-stop. I had no money … so I asked for a hundred baths (4 dollars) from each grandchild. … Next door sometimes also helps. So, I could go to see the doctor. (P6)*

COVID-19 also had financial impacts on participants’ lives. Participants found they had to be thrifty with money because they had no income, and they tried to manage money wisely. They did not spend money on unnecessary things.

*I want to get a job … doing something (to earn a living) like this. If not, I feel frustrated. When someone hired me, and I couldn’t go, (due to my illness and COVID-19) … so there is no income at all. (P3)*

## 4. Discussion

This study depicts the lived experiences of older persons with lymphoma undergoing chemotherapy during the COVID-19 pandemic. The description is organized under van Manen’s five lived lifeworlds. For our participants, lymphoma was a fatal disease and caused great suffering. In addition, chemotherapy treatments and their adverse effects impacted their daily living. In addition, COVID-19 and the participants’ comorbid illnesses created difficulties in their lives. The participants felt stressed and suffered from fear of death.

Thumjai is a cultural concept which reflects making up one’s mind to accept the truth in life. According to Mill et al. [34], thumjai is a Thai coping strategy which is typically performed in various phases of life. Its characteristics include accepting and letting go of unpleasant circumstances, discarding unpleasant emotions, steadying or easing the mind, and strengthening endurance and comprehension. Participants in this study believed that their cancer cannot be cured and that dying with suffering is the end point. Cancer is considered a fatal disease and dying with suffering is common for those who have it. Therefore, the participants tried to thumjai this life event. Thumjai reflected participants’ coping strategy to adapt to cancer and its impacts. For our participants, thumjai meant that they should not overthink or dwell on negative thoughts. They used Buddhist teaching to explain their life events, as Buddhist teaching helps them accept death.

The mode of thumjai processing used by our participants is similar to that reported by Khaw et al. [35], who found that Thai Buddhist older adults with advanced chronic organ failure accepted death through understanding it to be a natural part of life. They accepted that death is inevitable and were facing death with no fear, letting go of life and their earthly existence. In addition, Suwannapong [36] found that death acceptance among Thai patients with cancer was at high levels. Furthermore, the findings of our study support those of previous studies on emotional self-care. Thumjai worked as an emotional coping strategy for the participants. Emotional self-care aided them in managing all of their negative feelings and thoughts associated with their diagnosis, as well as helping them to prepare for the therapy and any adverse effects [37]. Thumjai brought them back into present time and helped them to focus more on living.

Continuing to fight cancer reflected the participants’ commitment to themselves. The older adults that participated in this study motivated themselves to keep fighting cancer. Despite suffering from adverse effects of chemotherapy treatments, they deliberately performed self-care by nourishing themselves, exercising, preventing infection, and being mindful of negative thoughts. Continuing to fight cancer reflected the participants’ aim to be self-reliant. The older participants’ perception that they were able to keep fighting cancer was strongly linked with their sense of control over the circumstances around them. Our findings support those of Corbett [38], who found that older adults with cancer expressed a sense of obligation for managing their disease and a desire to have personal control over their health.

To keep fighting cancer, participants relied on healthcare providers’ advice and their family’s support. The people with cancer interviewed in our study felt safe in the capable and trustworthy hands of healthcare professionals [39]. Participants considered healthcare providers to be significant in their cancer treatments. They entrusted their life to medical doctors and nurses. Good relationships with healthcare providers created a sense of hope for them. The participants complied with medical doctors and nurses’ instructions on self-care practices and self-monitoring.

Furthermore, family also played a crucial role in participants’ cancer journey. In Thai culture, keeping family ties is important and highly valued. In our study, participants said that they received help and support from their family members, including children, spouses, and relatives. This support made them feel loved and encouraged them to keep fighting cancer. However, most were hesitant to ask their children for help. They were concerned that they would be a burden to their children. As a result, they attempted to be independent and care for themselves well. In addition, participants were aware that they were at risk of contracting COVID-19. These participants mainly stayed at home and lived cautiously. They avoided going outside to prevent infections. Adhering to the preventive measures shared as part of public education during the pandemic helped them stay safe from COVID-19.

Karma guides the lives of Buddhist Thais. According to van Manen’s perspective on material or lived things, such things have a way of bringing people down or making people feel guilty about themselves. In our study, by using the lived things lens, karma disappointed participants or reflected their disappointment back to them. The participants’ acceptance of their current circumstances and death can be triggered by their belief in karma. They believed that previous misconduct was the root of their illness. The cancer diagnosis and their suffering from cancer treatment stimulated them to recall their previous misconduct. As a result, they had to thumjai and accept the consequences of their bad deeds or bad karma. Thinking in this way helped them compensate for the suffering they were experiencing. This can reflect self-care for their spirituality.

From a religious standpoint, giving an illness meaning helps one cope with it better. It supports a person’s ability to get through challenging times during treatment and appreciate their closeness to family, how they view their own life, and their faith in a brighter future for themselves and those around them [40]. Hence, religious faith aids in managing illness by giving patients feelings of inner peace, strength, guidance, and support, all of which facilitate self-care and caring for others [40]. Making merit (almsgiving to Buddhist monks) or other religious practices frequently revealed attitudes regarding karma and self-care among patients with terminal cancer [41]. In addition, praying and practicing spirituality can help people cope with their negative feelings and enhance their quality of life [42].

Our study highlighted the role of religion in the participants’ cancer journeys. Religious faith plays a significant role in Thais’ everyday lives. In our study, Thai older adults with lymphoma mobilized their religious belief to make meaning of their illness and to accommodate suffering. Participants applied the Buddhist principles of “the three characteristics of existence,” i.e., suffering, impermanence, and non-self, to comprehend their life events. In accordance with the present results, previous studies in several countries have demonstrated that religion provides moral and emotional support to patients throughout the process of coping with cancer [42,43]. These results confirm the association between religiosity and well-being [43,44,45]. Given the growing interest in spiritual accomplishments and the purpose of life, religion and spirituality may be especially relevant to the care of elderly cancer patients [46].

Cancer is considered a costly disease which can lead to financial hardships [47]. Participants reported difficulties in living after developing cancer and even more so during the COVID-19 pandemic. Due to COVID-19, patients with cancer reported having lower household incomes and expressed concern about their capacity to pay for cancer treatment [48]. Some participants said that they were too weak to continue to work. Also, social distancing made it harder for them. All participants were staying at home and were unemployed. They had to live their lives economically. They tried to pay for only necessary items and sought financial support from their families and networks, which also made them feel guilty about their families having to provide for them. Similar findings were reported by Sae, Abu El-Kas et al. [49], who concluded that financial hardship is common among patients with cancer. Financial issues had an impact on daily living, psychological health, and accessibility to care among patients with cancer during COVID-19.

Our results highlighted a number of issues that should be taken into account when implementing future interventions for older persons with lymphoma to ensure that they can access the interventions. Religion plays an important role in patients’ adaptation to cancer and interventions based on individual faith will be beneficial. Furthermore, older adults with lymphoma attempt to be self-reliant and rely on health information from healthcare professionals. Nurses can provide health education related to symptom management and self-care to older adults and their families. It has been suggested by older adults receiving cancer treatments that healthcare providers talk more slowly, use fewer medical terms, and give written information in addition to face-to-face contact with older people [50]. In addition, financial hardship is commonly reported among patients with cancer, and the COVID-19 pandemic created a chaotic situation affecting family income. It is crucial to strengthen multidisciplinary teamwork. Enhancing the roles of other professionals, such as social workers to address financial hardship and religious representatives for developing religiously tailored interventions, is recommended, as is exploring the role of community-based interventions in cancer care.

### Limitation

This study illustrated the specific lived experiences connected to self-care of older adults with lymphoma during the COVID-19 pandemic. These experiences were influenced by beliefs rooted in Buddhist doctrine and practices in the participants’ daily lives, thereby perhaps constraining non-religious philosophies. Furthermore, the themes in each lifeworld demonstrated interconnection, which can be understood as a holistic approach to health. The study sample was small, and the findings need to be explored with additional older adults and those with other faiths.

## 5. Conclusions

The self-care experiences of older adults with lymphoma during the COVID-19 pandemic highlighted the importance of cancer patients engaging in self-care practices to alleviate their suffering as their illness progresses and they undergo treatment. The older persons with lymphoma in our study tried to adapt to their illness by seeking spiritual support derived from their religious beliefs. Thumjai, as a form of emotional self-care, helped them calm down and stabilize their minds. They also deliberately performed self-care to fight cancer. Support from healthcare providers and their families played a crucial role in promoting independent living and mental well-being. During COVID-19, self-care was focused on maintaining safety at home. A spiritual–psychological intervention with a multidisciplinary team is recommended for further studies. Healthcare systems should support older cancer patients during the chaotic time caused by their emerging or re-emerging disease.

## Figures and Tables

**Table 1:** The demographic characteristics of the participants.

No	Sex	Age (Years)	Status	Cycle of Chemotherapy	Comorbidities	Caregivers
1	Male	62	Married	5	HT *, ICH *	Spouse
2	Male	66	Married	6	None	Older sister
3	Male	64	Single	6	HT, DLP *	Older sister
4	Male	72	Married	7	IHD *	Son/Daughter
5	Male	66	Married	2	Stroke	Spouse
6	Female	63	Single	2	HT	Son/Daughter
7	Male	67	Married	13	HT, DLP *	Son/Spouse
8	Female	66	Married	5	HT, DM *, DLP *	Son/Daughter
9	Female	65	Married	6	HT *	Son/Daughter

* HT = hypertension; * ICH = intracerebral hemorrhage; * IHD = ischemic heart disease; * DLP = dyslipidemia; * DM = diabetes mellitus.

**Table 2:** Subthemes and description based on 5 lifeworld existentials.

Lived World	Sub-Themes	Description
1. Lived body	Thumjai	Thumjai is a Thai word reflecting an approach that participants use to cope with their current illness and its impacts. Thumjai means accepting one’s own destiny and living in the present.
	Continuing to fight cancer	An approach to maintain self-independence. Participants tried to nourish their body with food and perform exercise.
2. Lived relation	Trusting healthcare providers	Participants adhered to healthcare providers’ recommendations with the belief that they could recover from illness.
	Holding on together	Participants cared for the minds of their families and relatives. They were aware that if they got worse, they would be a burden to others. Also, they received encouragement from their family and significant others, which inspired them to live.
3. Lived time	Being aware of the natural path of life	One’s lifetime is predetermined and unpredictable. One has to live one’s life consciously.
4. Lived space	Staying safe at home and staying cautious	Participants were aware that they were a high-risk group for COVID-19. They mainly stayed at home and were cautious when going out.
5. Lived things	Accepting one’s own karma	Participants viewed lymphoma as a karmic disease. They had to compensate for bad karma they had accumulated through merit-making and doing good deeds.
	Living economically	COVID-19 affected family economies. Participants could not work and had no income. They had to live lives economically.

## Data Availability

The data presented in this study are not available due to privacy and ethical restrictions.
